# Establishment of a novel hepatocyte model that expresses four cytochrome P450 genes stably via mammalian-derived artificial chromosome for pharmacokinetics and toxicity studies

**DOI:** 10.1371/journal.pone.0187072

**Published:** 2017-10-24

**Authors:** Daisuke Satoh, Satoru Iwado, Satoshi Abe, Kanako Kazuki, Shinobu Wakuri, Mitsuo Oshimura, Yasuhiro Kazuki

**Affiliations:** 1 Chromosome Engineering Research Center, Tottori University, Tottori, Japan; 2 Department of Biomedical Science, Institute of Regenerative Medicine and Biofunction, Graduate School of Medical Sciences, Tottori University, Tottori, Japan; 3 Food and Drug Safety Center, Tokyo, Japan; University of Kentucky, UNITED STATES

## Abstract

The utility of HepG2 cells to assess drug metabolism and toxicity induced by chemical compounds is hampered by their low cytochrome P450 (CYP) activities. To overcome this limitation, we established HepG2 cell lines expressing major CYP enzymes involved in drug metabolism (CYP2C9, CYP2C19, CYP2D6, and CYP3A4) and CYP oxidoreductase (POR) using the mammalian-derived artificial chromosome vector. Transchromosomic HepG2 (TC-HepG2) cells expressing four CYPs and POR were used to determine time- and concentration-dependent inhibition and toxicity of several compounds by luminescence detection of CYP-specific substrates and cell viability assays. Gene expression levels of all four *CYPs* and *POR*, as well as the CYP activities, were higher in TC-HepG2 clones than in parental HepG2 cells. Additionally, the activity levels of all CYPs were reduced in a concentration-dependent manner by specific CYP inhibitors. Furthermore, preincubation of TC-HepG2 cells with CYP inhibitors known as time-dependent inhibitors (TDI) prior to the addition of CYP-specific substrates determined that CYP inhibition was enhanced in the TDI group than in the non-TDI group. Finally, the IC_50_ of bioactivable compound aflatoxin B1 was lower in TC-HepG2 cells than in HepG2 cells. In conclusion, the TC-HepG2 cells characterized in the current study are a highly versatile model to evaluate drug-drug interactions and hepatotoxicity in initial screening of candidate drug compounds, which require a high degree of processing capacity and reliability.

## Introduction

Primary human hepatocytes are recommended for use in studies to predict hepatic drug metabolism and hepatotoxicity of pharmaceutical candidate compounds [1, 2]. However, primary human hepatocytes are not only expensive and ethically problematic but also are challenging to conduct long-term assays due to their short lifespan and differences between lots; therefore, establishing specific evaluation criteria for candidate compounds remains challenging [3, 4]. These issues highlight the need for alternative human liver derived cell lines that can be used for general pharmacokinetics and hepatotoxicity evaluation studies [5]. Currently available alternative *in vitro* models utilizing a human liver cancer cell line HepG2 is used to provide supplementary data to support findings obtained from human hepatocytes [6, 7]. However, the expression levels of drug-metabolizing enzymes are significantly lower in the cell line than in primary human hepatocytes *in vitro* [8–13]. Due to these concerns, HepG2 cell lines over expressing cytochrome P450 (CYP)3A4 and related enzymes were developed [14–19].

Gene transduction can be achieved by two major methods. Transient transfection utilizes a plasmid vector [16, 17] or an adenoviral vector [14] to introduce target gene to the cell. The major advantage of this method is its ease of use in *in vitro* studies; however, inability to accurately control the number of copies introduced into each cell, which leads to wide variations of experimental data, is a major caveat. As an alternative, stable gene expression is achieved by insertion of the gene of interest into host chromosome using retroviral [9, 11, 15, 19] or PiggyBac vectors [18]. However, this approach leads to the destruction of host genomes. Furthermore, long-term culturing causes epigenetic silencing of host chromosomes, again leading to wide variations in the level of gene expression. Thus, cell-based systems reported by one research group via conventional gene transduction methods may not be reproducible by other groups.

We developed mammalian-derived artificial chromosomes [20, 21], which were derived from a native mouse chromosome (designated as MAC). MAC harbors no endogenous genes and contain loxP site for gene loading and green fluorescent protein (*GFP*) for monitoring its presence [22, 23]. The prominent advantage of MAC as gene delivery vector is no limitation of DNA insert size, which allows the introduction of all required genes. The mitotic stability of the MAC vectors with fixed copy number of gene permits to reduce clonal variation of the gene expression levels. Semipermanent retention of gene/s of interest in host cells allows for the construction of sustainable models. In addition, because MAC vectors are independently maintained without disruption of host chromosomes, gene transduction can proceed without disrupting the host functions. Thus, the aim of this study was to establish a novel HepG2-based cell line that stably expressed multiple pharmacokinetic-related genes lacking in parental HepG2 cells by using MAC vectors carrying four kinds of CYPs and POR genes to evaluate hepatic drug metabolism and hepatotoxicity.

## Materials and methods

### Chemicals

Erythromycin, clarithromycin, roxithromycin, diltiazem, ritonavir, ketoconazole, fluconazole, cimetidine, ticlopidine, omeprazole, fluvoxamine, paroxetine, fluoxetine, quinidine, aflatoxin B1, and sterigmatocystin were obtained from Wako, Japan. Gestodene and mifepristone were obtained from Tokyo Chemical Industry, Japan. G418 was obtained from Funakoshi, Japan. Ethinylestradiol, rifampicin, tienilic acid, terbinafine, hypoxanthine, aminopterin, and thymidine (HAT) and 4,6-diamidino-2-phenylindole (DAPI) were obtained from Sigma Aldrich, USA. All other chemicals were of the highest grade commercially available.

### Construction of the 4CYPs-POR gene-loading PAC vector

First, we used artificial gene synthesis to develop a gene-loading PAC vector containing all four *CYP*s and *POR* (Invitrogen). A gene modification method using a P1-derived artificial chromosome (PAC) vector was performed, as described in our previous report [24] and detailed in S1A Fig. cDNA of four *CYPs* expressed in human hepatocytes (*CYP2C9*, *CYP2C19*, *CYP2D6*, *CYP3A4*) [25] and CYP oxidoreductase (*POR*) [26] were obtained as artificial synthetic genes (Invitrogen). Sequence data for all cDNAs were obtained from the UCSC Genome Browser (https://genome.ucsc.edu/). Construction of the basic framework was initiated by introduction of the cDNAs downstream of the forced expression promoter CAG. Next, insulators (HS4) containing a sequence to stabilize gene expression were ligated to flank the genes. The CAG-CYP and CAG-POR plasmids were digested with SalI and AvrII and inserted to the SalI/AvrII site of the HS4-carrying plasmid for the construction of four kinds of HS4-CAG-CYP and HS4-CAG-POR plasmids. Next, the HS4-CAG-CYP2C9 plasmid was digested with NheI/AscI site and ligated with the HS4-CAG-CYP2C19, which was digested with AscI/AvrII, for the construction of HS4-CAG-CYP2C19-HS4-CAG-CYP2C9 plasmid. Similarly, the HS4-CAG-CYP2D6 plasmid was digested with NheI/AscI and ligated with the HS4-CAG-CYP3A4, which was digested with AscI/AvrII, for the construction of HS4-CAG-CYP3A4-HS4-CAG-CYP2D6 plasmid. Then, the HS4-CAG-CYP2C19-HS4-CAG-CYP2C9 and the HS4-CAG-CYP3A4-HS4-CAG-CYP2D6 plasmids were digested with AscI/AvrII and ligated in sequence to the HS4-HPRT-loxP-PAC vector, which was digested with NheI/AscI, for the construction of the PAC vector containing all four *CYP*s. Finally, HS4-CAG-POR plasmid was inserted to the PAC vector containing all four CYPs by the same technique mentioned above (S1A Fig). At all stages of the construction of the gene-loading PAC vector, PCR analysis was used to confirm that all target genes were loaded in sequence (S1B Fig).

### Cell culture

A MAC6 vector was used to generate the 4CYPs-POR MAC. The structure of MAC6 contained a centromere from mouse chromosome 11, EGFP flanked by HS4 insulators, PGKneo, loxP-5’HPRT site, PGKpuro, and telomeres (S2 Fig) [27]. Hypoxanthine phosphoribosyl transferase (HPRT)-deficient Chinese hamster ovary (CHO; JCRB0218) hybrids containing only the MAC6 or the 4CYPs-POR MAC were maintained in Ham’s F-12 nutrient mixture (Invitrogen, Carlsbad, CA, USA) supplemented with 10% fetal bovine serum and 600 μg/mL G418. HepG2 cells (HB-8065, ATCC, USA) were maintained in Dulbecco’s modified Eagle’s medium (Sigma-Aldrich) supplemented with 10% fetal bovine serum, non-essential amino acids (Wako), pyruvate (Wako), and penicillin-streptomycin (Wako).

### Microcell-mediated chromosome transfer (MMCT)

Measles virus envelope protein-mediated-MMCT (MV-MMCT) was performed as described previously [22, 28]. CHO cells containing 4CYPs-POR MAC and expressing MV-H and MV-F proteins were used as donor microcell hybrids. We transiently expressed the MV-H and MV-F in CHO cells with the 4CYPs-POR MAC. Next day, the cells were transferred into flasks, and after incubation overnight, micronuclei formation in CHO cells was induced by 72-hours treatment with 0.1 μg/mL colcemid. The cells were centrifuged with cytochalasin B to isolate microcells. Next, the collected microcells were cocultured with HepG2 cells for the fusion of micronuclei with the recipient cells. To select for HepG2 cells containing the 4CYPs-POR MAC, cells were cultured in media containing G418 (600 μg/mL). A few clones of HepG2 colonies appeared in each 100-mm dish. The distance between GFP-positive colonies on the culture dishes was sufficiently great to enable us to determine single colonies by visual inspection under a microscope. The colonies were dense, and there appeared to be no contamination with other colonies. The clones used for the tests in this study were derived from colonies not from the same culture dish but from different culture dishes. In each line, 4CYPs-POR MAC-transferred HepG2 cells were characterized by PCR and fluorescence *in-situ* hybridization (FISH) analyses as follows.

### Fluorescence *in situ* hybridization (FISH)

FISH analyses were performed using spreads of fixed chromosomes in either metaphase or interphase from each cell hybrid, a digoxigenin-labeled (Roche, Switzerland) mouse *cot-1* DNA probe (Invitrogen), and a biotin-labeled 4CYPs-POR PAC probe. Chromosomal DNA was counterstained with DAPI. Images were captured using an AxioImagerZ2 fluorescence microscope (Carl Zeiss GmbH).

### Genomic polymerase chain reaction (PCR)

Genomic DNA was extracted from cell lines using a genomic DNA extraction kit with DNase-free RNase (Gentra Systems, Minneapolis, USA). Primer sequences are listed in Table 1. Primers used for amplification of the 4CYPs-POR transgene spanned the intron and did not amplify any products in host genomic DNA.

**Table 1:** Primer sequences.

Sets	Primers	Sequences (5' to 3')	Notes
1	gPCR hCYP2C9_F	ATGGATTCTCTTGTGGTCCT	Vector construction check by PCR
	gPCR hCYP2C9_R	TCAGACAGGAATGAAGCACA	
2	gPCR hCYP2C19_F	ATGGATCCTTTTGTGGTCCT	Vector construction check by PCR
	gPCR hCYP2C19_R	TCAGACAGGAATGAAGCACA	
3	gPCR hCYP2D6_F	ATGGGGCTAGAAGCACTGGT	Vector construction check by PCR
	gPCR hCYP2D6_R	CTAGCGGGGCACAGCACAAA	
4	gPCR hCYP3A4_F	ATGGCTCTCATCCCAGACTT	Vector construction check by PCR
	gPCR hCYP3A4_R	CTCAGGCTCCACTTACGGTG	
5	gPCR hPOR_F	ATGATCAACATGGGAGACTC	Vector construction check by PCR
	gPCR hPOR_R	CTAGCTCCACACGTCCAGGGA	
6	qPCR hCYP2C9_F	CCACATGCCCTACACAGATG	Gene expression analysis by real-time RT-PCR
	qPCR hCYP2C9_R	TGCCCTTGGGAATGAGATAG	
7	qPCR hCYP2C19_F	ACTTGGAGCTGGGACAGAGA	Gene expression analysis by real-time RT-PCR
	qPCR hCYP2C19_R	CATCTGTGTAGGGCATGTGG	
8	qPCR hCYP2D6_F	TGATGAGAACCTGcGCATAG	Gene expression analysis by real-time RT-PCR
	qPCR hCYP2D6_R	CCCTATCACGTCGTCGATCT	
9	qPCR hCYP3A4_F	TGTGGGGCTTTTATGATGGT	Gene expression analysis by real-time RT-PCR
	qPCR hCYP3A4_R	CCTCCGGTTTGTGAAGACAG	
10	qPCR hPOR_F	TGGAGGAGGACTTCATCACC	Gene expression analysis by real-time RT-PCR
	qPCR hPOR_R	ACAAGCTCGTACTGGCGAAT	
11	qPCR Nat1_F	ATTCTTCGTTGTCAAGCCGCCAAAGTGGAG	Gene expression analysis by real-time RT-PCR
	qPCR Nat1_R	AGTTGTTTGCTGCGGAGTTGTCATCTCGTC	

gPCR: Genomic polymerase chain reaction; qPCR; quantitative PCR hCYP; human cytochrome P450 POR; CYP oxidoreductase

### mRNA preparation and reverse transcription (RT)–quantitative PCR (qPCR) analysis

mRNAs were extracted using the RNeasy Mini Kit (Qiagen, Germany), according to the manufacturer’s instructions. First-strand cDNA synthesis was performed using an oligo-(dT) 20-mer primer and the SuperScript III reverse transcriptase (Invitrogen), according to the manufacturer’s instructions. Primer sequences are listed in Table 1. qPCR was performed using the Power SYBR Green PCR Master Mix (Applied Biosystems) on an ABI Step One Plus device (Applied Biosystems). *NAT1*, a housekeeping gene, was used as control.

### Assessment of drug metabolism

Measurement of the activity of all four CYPs was conducted using the P450-Glo^™^ assay (Promega, Japan), according to the manufacturer’s instructions. Briefly, cells were seeded in a 96-well plate at a density of 1×10^4^ cells/well. Twenty-four hours later, test compounds and the luminescent substrate included in the kit were added into the culture media. Following 1 hour of incubation, the detection reagent was added, and the luminescence generated by the metabolic substrate within the culture media was measured using an Infinite F500 plate reader (Wako). The inhibitor test conditions utilized in this study are listed in the results.

### Assessment of hepatotoxicity

Hepatotoxicity of test compounds was determined by the ratio of surviving cells using the neutral red (NR) uptake assay (Kanto Chemical Co., Inc., Japan). Briefly, cells were seeded in a 96-well plate at a density of 1×10^4^ cells/well. After 24 hours, test compounds were added for an additional 48 hours. At the end of treatments, cells were washed with Dulbecco's phosphate-buffered saline (Sigma-Aldrich), and NR-containing medium was added for an additional 3 hours. Following the removal of the NR medium, cells were washed, and NR was extracted. Optical density of wells was measured at 540 nm with a Sunrise Classic microplate reader (TECAN, Austria).

### Statistical analysis

Curve fitting was performed using the SigmaPlot 13.0 statistical software (Systat Software, IL, USA). To determine compound concentrations to achieve 50% of maximum effective or inhibitory response (IC_50_, respectively), concentration-response data were fitted into a four-parameter sigmoidal (Hill) function. Data were presented as means ± S.D. Statistical analyses were carried out using unpaired Student’s *t* test. The level of statistical significance was set at *P*< 0.05.

## Results

### Construction of the MAC vector containing 4CYPs and POR

**Fig 1:**
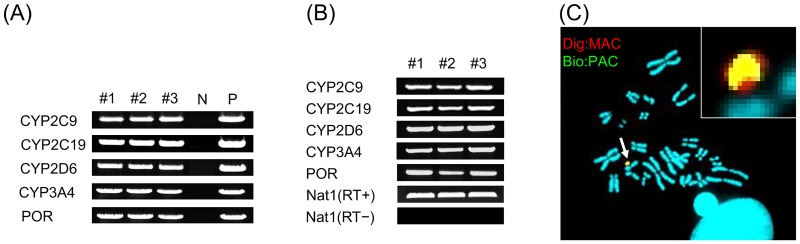
Construction and analysis of the 4CYPs-POR MAC vector in CHO cells. (A) Genomic PCR and (B) RT-PCR analysis of CHO cells containing the 4CYPs-POR MAC. N, negative control (parental CHO cells); P, positive control (PAC-4CYPs-POR). (C) FISH analysis of donor CHO cells containing the 4CYPs-POR MAC. Digoxigenin-labeled mouse *cot-1* DNA (red) was used to detect the MAC. Biotin-labeled 4CYPs-POR PAC (green) was used to detect the 4CYPs-POR cassette in the MAC. Chromosomal DNA was counterstained with DAPI. White arrow indicates MAC vector, and the inset shows enlarged image of the MAC.

CHO cells containing the empty MAC vector were transiently transfected with a Cre expression vector and the 4CYPs-POR PAC vector, as mentioned above (S2 Fig). The transfected cells were passaged the next day and were cultured with the selection medium containing HAT. Ultimately, a total of 40 HAT-resistant CHO cell clones carrying the MAC vector with the transgenes were obtained. Analysis by RT—qPCR confirmed the expression of *CYP2C9*, *CYP2C19*, *CYP2D6*, *CYP3A4*, and *POR*, and representative three clones were selected. Genomic PCR analyses detected all transgenes in three clones (Fig 1A). We determined that all three clones exhibited high levels of expression for all four *CYPs* and *POR* (Fig 1B), as compared to the parental CHO cells that did not express any of the genes.

FISH analysis indicated that the MAC was independently maintained in these three CHO clonal cell lines (Fig 1C). Because we were also able to observe signals indicating the construct we introduced into the MAC, we were able to confirm that the desired MAC, designated as 4CYPs-POR MAC, was successfully constructed.

### Transfer of the 4CYPS-POR MAC vector into HepG2 cells

**Fig 2:**
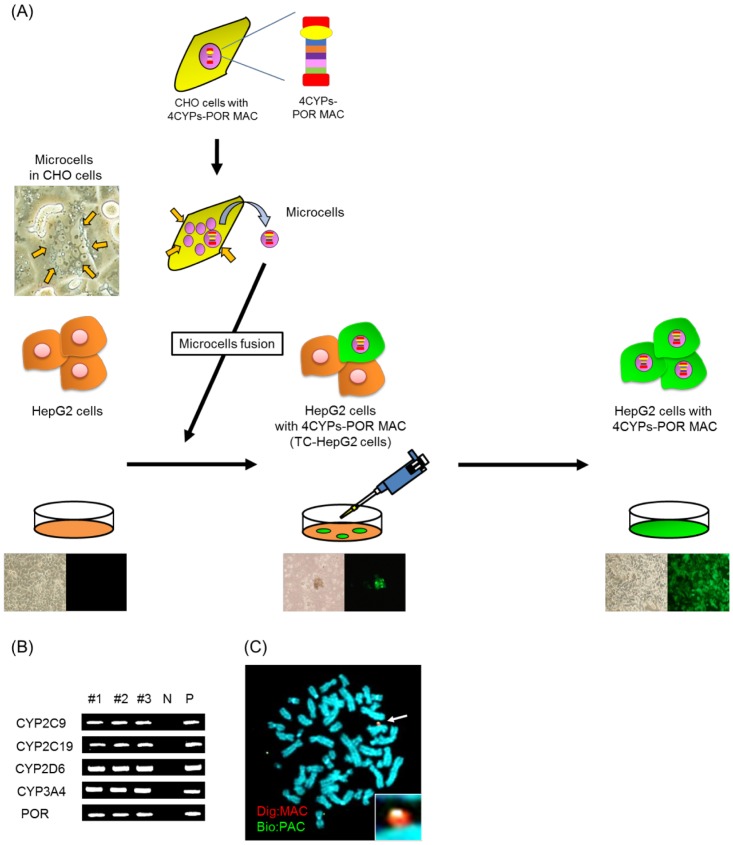
Analysis of HepG2 cells containing the 4CYPs-POR MAC. (A) Flowchart of the MAC transfer from donor CHO cells to recipient HepG2 cells via MMCT method, which comprises the following steps: micronucleation of donor cells by colcemid, enucleation by cytochalasin B, and microcell purification and fusion with recipient HepG2 cells. HepG2 hybrids were selected with 400 μg/mL G418 and picked for clonal expansion. (B) G418-resistant clones are screened by genomic PCR to determine the presence of the 4CYPs-POR transgene. (C) Representative metaphase fluorescence *in situ* hybridization images of TC-HepG2 cells. Digoxigenin-labeled mouse *cot-1* DNA (red) was used to detect the MAC. Biotin-labeled pPAC-4CYPs-POR (green) was used to detect the 4CYPs-POR cassette in the MAC. Chromosomal DNA was counterstained with DAPI. White arrows indicate MAC vectors, and the inset shows an enlarged image of the MAC.

**Fig 3:**
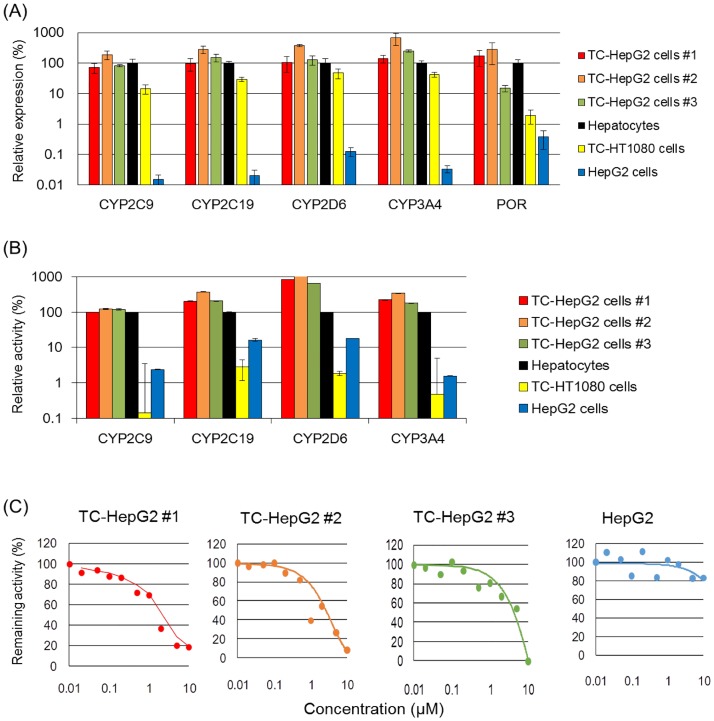
Analysis of CYP expression and activity in TC-HepG2 cells. (A) Reverse transcription-qPCR analysis for the expression of CYPs in TC-HepG2 cells. Each column represents the mean ± S.D. (*n* = 3). (B) CYP activity analysis in TC-HepG2 cells. The expression and activity levels for each CYP relative to the levels in human primary hepatocytes are shown. Each column represents the mean ± S.D. (*n* = 3). (C) CYP3A4 inhibition activity in TC-HepG2 cells determined by clarithromycin.

MV-MMCT was performed to transfer 4CYPS-POR MAC from CHO cells into HepG2 cells, as described previously [28]. Eight GFP+/G418-resistant HepG2 clones were obtained. We randomly picked up three out of the eight GFP+/G418-resistant clones for following analyses. Genomic PCR determined that all of these clones expressed *CYP2C9*, *CYP2C19*, *CYP2D6*, *CYP3A4*, and *POR* genes (Fig 2B). Donor CHO cells were used as the positive control for genomic PCR, whereas the parental HepG2 cells were used as the negative control. FISH analysis of these three clones revealed that the 4CYPs-POR MAC was independently maintained in the HepG2 cells without integration or translocation into host chromosomes (Fig 2C). The HepG2 cells containing a single copy of 4CYPs-POR MAC were defined as transchromosomic-HepG2 (TC-HepG2) cell clones. Genes specifically expressed in liver cells [i.e., albumin (ALB) and tyrosine aminotransferase (TAT)] and a gene expressed in liver cancer/immature cells [i.e., α-fetoprotein (AFP)] were used as endogenous control genes. None of them was affected by either inter-clone competition. In contrast, RT—qPCR revealed that the expression levels of all five genes were higher in the TC-HepG2 cells than the parental HepG2 cells. Furthermore, the expression levels of these genes in the TC-HepG2 cells were similar to those in primary human hepatocytes, and there was minimal variation in transgene expression levels between the different clones (Fig 3A). Finally, the assessment of the metabolic activity of four CYPs indicated that all three clones were more active than the original HepG2 cells (Fig 3B) and that the CYP activity was either comparable or higher than those in primary human hepatocytes. In addition, we introduced the 4CYPs-POR MAC into HT1080 cells, which are human-derived non-hepatic cell lines. Consequently, although the gene expression increased, CYP activity was barely detectable. Variation between the TC-HepG2 clones, which was within the range of three folds, was minimal. Assessment of the inhibitory effect of clarithromycin [29] on CYP3A4 in three TC-HepG2 cells indicated that there was a concentration-dependent inhibitory effect on metabolic activity (Fig 3C). Overall, these results indicated that the 4CYPs-POR MAC was functional in the TC-HepG2 cells.

### Stability of the 4CYPs-POR MAC and five transgene expression in TC-HepG2 cells

**Fig 4:**
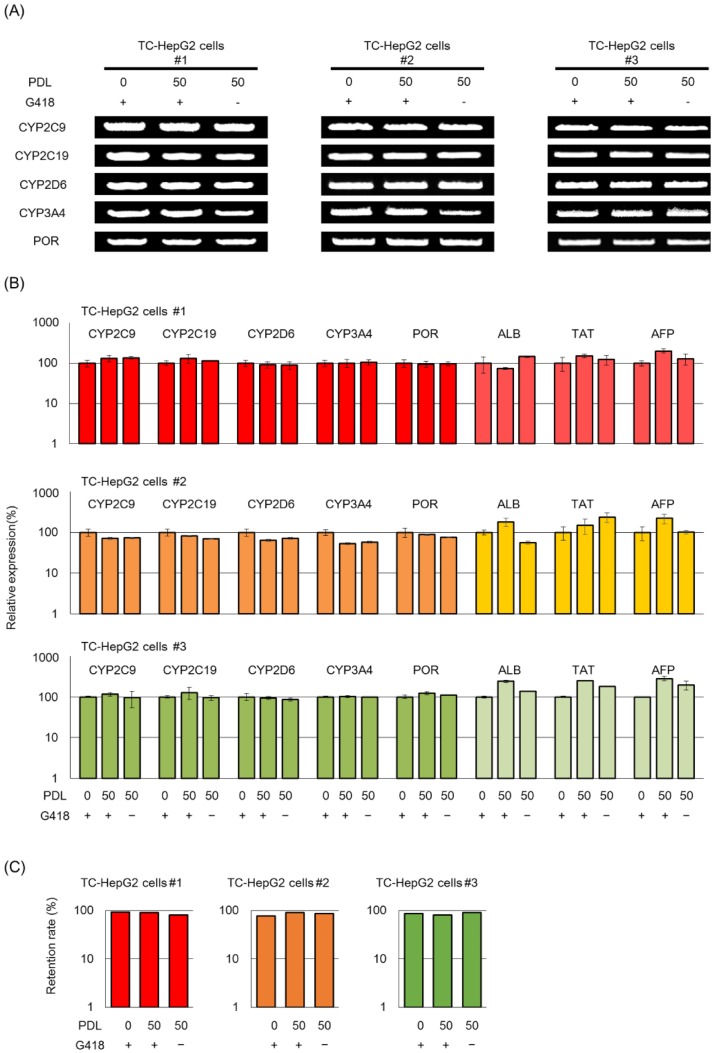
The 4CYPs-POR MAC stability and sustained transgene regulation in the TC-HepG2. TC-HepG2 cells were used for the following analyses before and after long-term culturing (50 PDLs) in the presence and absence of selection with G418 (A-C). (A) Genomic PCR analysis of the 4CYPs-POR MAC in TC-HepG2 cells. (B) RT-qPCR analysis for expression of the transgenes and endogenous genes in TC-HepG2 cells. The 4CYPs-POR transgene expression levels at 50 PDLs are standardized to *NAT1* expression, and the relative each CYP and POR gene expression in each clone at 0 PDLs was used as control. Each column represents the mean ± S.E. (*n* = 3). (C) FISH analyses for 4CYPs-POR MAC stability in TC-HepG2 cells (*n* = 25–30). PDL, population doubling.

The TC-HepG2 cells were cultured until they reached 50 population doubling levels (PDLs), and the effect of drug selection (400 μg/mL G418) on the stability of the MAC and the 4CYPs-POR transgene expression was determined. First, stability of the 4CYPs-POR MAC during long-term culture conditions was assessed. The results indicated that the inserted genome was maintained in the presence and absence of the drug selection during long-term culturing (Fig 4A). Regarding endogenous gene expression (ALB, TAT, AFP), there was no change, regardless of long-term culture (Fig 4B). RT—qPCR revealed that there were no decreases in the expression level of the five transgenes on the MAC, indicating that stable gene expression was maintained during long-term culturing (Fig 4B). Even we cultured to 50 PDLs in the absence of selection drug G418, the expression levels for all genes were stable as well.

Because the loss of the 4CYPs-POR MAC from the TC-HepG2 cells was possible during long-term culturing, the retention rate of the MAC was determined using FISH analysis. The results indicated that the MAC was stably maintained within the cells even after long-term culturing (Fig 4C).

### Assessment of drug-drug interactions in TC-HepG2 cells

As the results thus far indicated that there was little variation among different TC-HepG2 clonal cell lines, the subsequent stability tests were performed using TC-HepG2 clone #1. Twenty compounds that are known to inhibit CYPs were used to calculate IC_50_ values in TC-HepG2 cells, which were then compared to those in primary human hepatocytes. The results clearly demonstrated that all tested compounds exerted inhibitory effects in TC-HepG2 cells. Furthermore, the inhibitory effects of these compounds in TC-HepG2 cells were comparable to those reported in primary human hepatocytes (Table 2). Overall, these results suggested that the TC-HepG2 cells successfully replicated their CYP-inhibitory effects observed in human hepatocytes.

**Table 2:** Summary of *in vitro* inhibition data for CYP3A4, CYP2C9, CYP2C19, and CYP2D6 for 20 compounds. Values represent means ±S.D. of triplicate determinations.

Target CYP	Drug	IC50 (μM)	
		TC-HepG2 cells	Hepatocytes
CYP3A4	Ethinylestradiol	0.97 ± 0.11	0.46 ± 0.05
	Gestodene	58.5 ± 4.6	7.4 ± 0.3
	Mifepristone	11.9 ± 4.5	6.0 ± 0.4
	Erythromycin	5.7 ± 1.7	3.4 ± 0.2
	Clarithromycin	5.9 ± 0.3	5.0 ± 0.2
	Roxithromycin	7.9 ± 0.2	9.9 ± 0.2
	Diltiazem	9.1 ± 4.1	7.4 ± 0.4
	Ritonavir	0.74 ± 0.03	1.0 ± 0.6
	Ketoconazole	0.067 ± 0.010	0.047 ± 0.005
	Fluconazole	1.98 ± 0.34	2.0 ± 0.2
	Cimetidine	41 ± 19	7.1 ± 0.3
	Rifampicin	131.4 ± 21.6	78 ± 12
CYP2C9	Ticlopidine	0.37 ± 0.03	0.35 ± 0.04
	Omeprazole	4.4 ± 0.5	8.4 ± 0.7
CYP2C19	Tienilic acid	5.7 ± 1.7	0.55 ± 0.02
	Fluvoxamine	0.0042 ± 0.0007	0.0030 ± 0.0004
CYP2D6	Paroxetine	10.9 ± 4.5	6.8 ± 0.7
	Fluoxetine	0.056 ± 0.049	0.044 ± 0.006
	Quinidine	0.0089 ± 0.0003	0.089 ± 0.003
	Terbinafine	0.0021 ± 0.0002	0.0099 ± 0.0002

CYP; cytochrome P450

**Fig 5:**
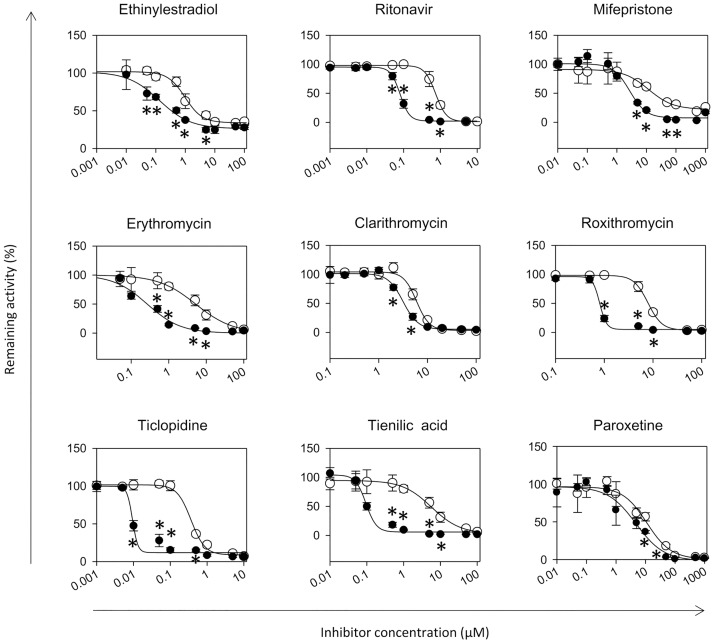
Relationship between relative CYP activity level and inhibitor concentration. Relative CYP activity was determined as the ratio of CYP-specific luminescence in the presence of the specific inhibitors to that in the absence of the same inhibitor. Open circles represent the values in cultures treated with the inhibitor and the CYP-specific luminescent substrate simultaneously [direct inhibition (DI)], and closed circles represent the values in cultures pretreated with the inhibitor for 60 min before the addition of the luminescent substrate (time-dependent inhibition). Values represent means ± S.D. (n = 4). *P < 0.05 vs each DI group.

Fig 5 shows the results of the experiments assessing the irreversible inhibitory response of the TC-HepG2 cells to different compounds. The inhibitors used in these experiments included CYP3A4 inhibitors ethinylestradiol [30], ritonavir [31], mifepristone [32], erythromycin [33], clarithromycin [29], and roxithromycin [29]; CYP2C9 inhibitor tienilic acid [33]; CYP2C19 inhibitor ticlopidine [34]; and CYP2D6 inhibitor paroxetine [35]. Briefly, CYP inhibitors were added to the culture media at the same time as the metabolic substrate to test direct inhibition (DI) or 1 hour prior to the addition of the metabolic substrate to test time-dependent inhibition (TDI). The inhibitory effects of the compounds were higher in TC-HepG2 cultures that were preincubated with the inhibitors than those that were incubated with the inhibitor and the substrate simultaneously. These results were consistent with previous studies and suggested that TC-HepG2 cells could be used for the detection of TDI *in vitro*.

**Fig 6:**
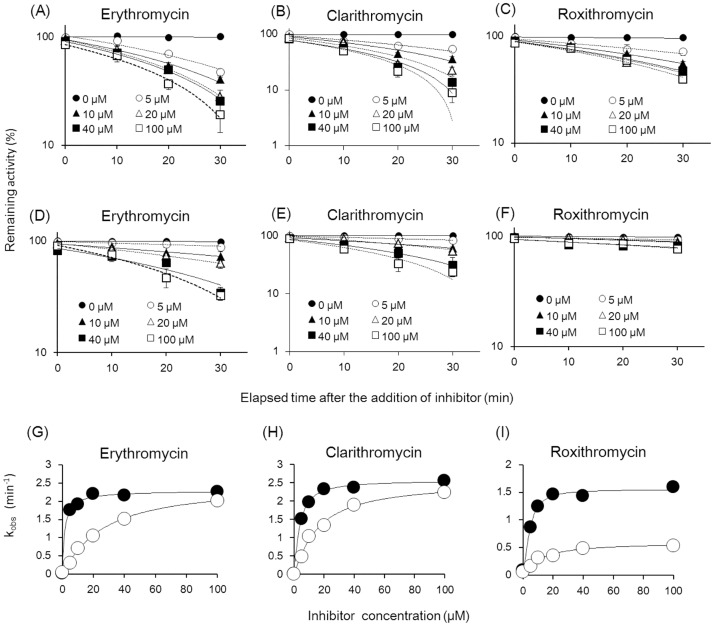
Inhibitory effects of macrolides on CYP-mediated metabolism in TC-HepG2 cells. (A), (B), (C) CYP3A-specific metabolism of clarithromycin, erythromycin, and roxithromycin, respectively, in TC-HepG2 cells. (D), (E), (F) CYP3A-specific metabolism of clarithromycin, erythromycin, and roxithromycin, respectively, in human hepatocytes. Macrolides (0–100 μM/L) were preincubated at 37°C for 0–30 min, and the reaction was initiated by the addition of CYP3A-specific substrate for 30 min. (G—I) Apparent inactivation rate (k_obs_) was estimated graphically from the slope of the plot for the natural log enzyme activity remaining after preincubation against preincubation time and was corrected for any loss of activity in the absence of the inhibitor. k_inact_ and K_i,app_ values were obtained from a double reciprocal plot of k_obs_ against inhibitor concentration. Closed circles represent TC-HepG2 cells, and opened circles represent parental HepG2 cells. Each symbol represents the average of triplicate determinations.

We also determined whether three inhibitors (erythromycin, clarithromycin, and roxithromycin), which induced mechanism-based inhibition (MBI) in humans, exerted similar effects in TC-HepG2 cells [29]. Specifically, increasing concentrations of the inhibitors (0, 5, 10, 20, 40, or 100 μM) were added 10, 20, or 30 min prior to the addition of the luminescent CYP3A4 substrate, and the luminescence levels in culture media were used to determine metabolite levels. Fig 6A–6F shows percent changes in luminescence in cultures treated with the inhibitors compared to those in control cultures not treated with the inhibitors. All three inhibitors suppressed the generation of metabolites in a time- and concentration-dependent manner. Among the three compounds, all macrolide antibiotics, the inhibitory effect of roxithromycin is lower than those of erythromycin and clarithromycin in humans. Accordingly, the inhibitory effect of roxithromycin in TC-HepG2 cells was lower than those of the other two inhibitors. Further, because the CYP3A4 activity level in TC-HepG2 cells was higher than that in human hepatocytes (Fig 3), the rate of metabolic reduction by the inhibitors may be detected with high sensitivity. Fig 6G–6I show the plots to determine the rate of inactivation (k_obs_) for the three inhibitors as depicted in Fig 6A–6F. Next, maximal inactivation (k_inact_) and concentration at 50% k_inact_ (K_i,app_) values were calculated as indicators of MBI, using the method by Taesotikul et al. (2011) [36]. The k_inact_ calculated based on the data presented in Fig 6G–6I showed that the inhibitory effect of roxithromycin was lower than those of the other two inhibitors (Table 3). In addition, for all compounds, K_i,app_, indicating CYP reactivation, was lower in TC-HepG2 cells than in human hepatocytes. This finding suggested that TC-HepG2 cells could be used as a model to determine MBI with higher sensitivity than human hepatocytes.

**Table 3:** Determination of k_inact_ and K_i,app_ for metabolism-dependent inhibition of CYP3A4 by macrolides. Apparent inactivation rates (k_obs_) were estimated graphically from the slope of the plot for the natural log of enzyme activity remaining after preincubation against preincubation time and were corrected for any loss of activity in the absence of the inhibitor. k_inact_ and K_i,app_ values were obtained from a double reciprocal plot of k_obs_ against inhibitor concentration. Data represent means ± S.D. of triplicate determinations.

	TC-HepG2 cells		Hepatocytes	
	k_inact_	K_i,app_	k_inact_	K_i,app_
Erythromycin	2.29 ± 0.14	1.53 ± 0.96	2.43 ± 0.24	25.4 ± 5.6
Clarithromycin	2.56 ± 0.08	3.76 ± 0.42	2.52 ± 0.29	16.0 ± 4.3
Roxithromycin	1.55 ± 0.06	4.60 ± 0.60	0.58 ± 0.08	13.1 ± 4.7

TC; Transchromosomic

### Assessment of metabolic toxicity in TC-HepG2 cells

**Fig 7:**
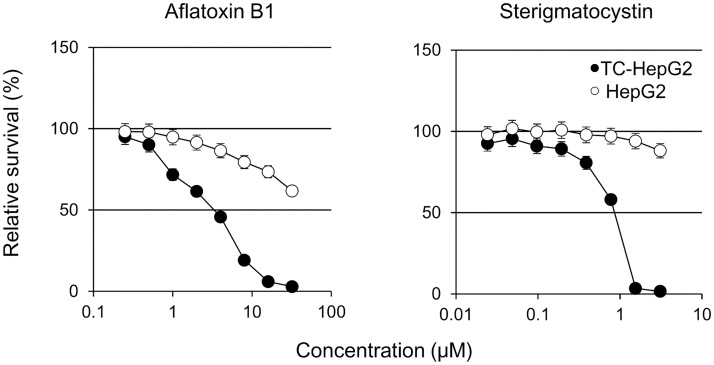
Assessment of compound-associated toxicity in TC-HepG2 cells. Aflatoxin B1 and sterigmatocystin requires CYP-mediated activation to form cytotoxic and DNA-reactive intermediates. Symbols represent means ± S.D. of triplicate determinations.

Next, we determined whether TC-HepG2 cells could be used to determine the metabolic toxicity of two compounds, aflatoxin B1 and sterigmatocystin. Both aflatoxin B1 and sterigmatocystin convert to active metabolites via CYP3A4 and exert hepatotoxicity through DNA damage [37, 38]. As shown in Fig 7, the cell survival rates were significantly lower in TC-HepG2 cells treated with either compound than in parental HepG2 cells under the same conditions. These results indicated that the TC-HepG2 cells could be used to assess not only hepatic metabolism but also hepatotoxicity of compounds associated with CYP-mediated metabolism.

## Discussion

Generation of highly reliable data requires the development of a highly reproducible model that produces few interfacility differences [3–5]. Several studies previously reported gene transfer into HepG2 cells using various methods [14–19]. However, due to the issues inherent to vector-based methods, even cells established at one research may not be reproducible by conventional gene transfer technologies at other facilities, which severely limits their utility in drug discovery research. Therefore, in the current study, we established HepG2 cell line using MAC vectors [20–23] to assess drug metabolism and toxicity as a reliable and reproducible system. Our findings indicated that one copy of the MAC with the 4CYPs-POR genes was transferred to parental HepG2 cells, which were detected alongside the host chromosomes. We also found that these transgenes were stably retained within the cells even after long-term culturing (Fig 4). In addition, because the copy numbers of the transferred genes could be controlled, there was minimal clonal variation (Fig 3). Moreover, transgene expression was maintained over the long-term culture conditions, and CYP activities derived from the transgenes were either comparable or higher than those detected in human hepatocytes. Altogether, these results indicated that the TC-HepG2 cell lines were exceptionally stable and exhibited a high degree of metabolic activity.

POR is a flavin enzyme that supplies electrons necessary for enzymatic reactions catalyzed by CYPs. When no genes were introduced, HepG2 cells exhibited low expression of POR and CYP. To complement the lack of expression, we incorporated the POR gene. In addition, we introduced the 4CYPs-POR MAC into HT1080 cells, which are human-derived non-hepatic cell lines. Consequently, although the gene expression increased, CYP activity was barely detectable. These results suggest that the introduction of other factors, in addition to POR as a coenzyme, is required to create substitutable hepatic cells from human non-hepatic cells for testing CYP metabolism.

One major reason for the dropout of candidate compounds during development the removal of novel drugs from the market after launch [39–45] is drug-drug interactions (DDIs) caused by CYP inhibition [46–48]. To avoid DDIs, pharmaceutical companies implement CYP inhibition studies starting with the initial drug discovery stage. CYP inhibition patterns are categorized as either DI or TDI [49]. Most of the DI actions of compounds are reversible; therefore, inhibitory effects of the administered drug itself diminish as it is removed from the body through metabolism and excretion. While, potential mechanisms for TDI include the formation of a tight-binding, quasiirreversible inhibitory metabolite complex or the inactivation of CYPs by covalent adduct formation, which is retained even after the administered drug itself is eliminated from the body. As a result, in clinical settings, TDI often leads to far more serious adverse effects than DI. Therefore, in early stages of drug discovery, clear distinctions between DI and TDI are necessary for accurate assessment of candidate compounds. In the current study, we performed inhibition studies in TC-HepG2 cells using existing medical compounds. The result demonstrating that the extremely high IC_50_ values were highly correlated than those determined in human hepatocytes suggested that the TC-HepG2 cells were a viable alternative to human hepatocytes. All four CYP molecular species could replicate the TDI reported by previous studies using human hepatocytes. In order to assess these DDIs in detail, we calculated the k_inact_ and K_i,app_ values as well. The results indicated that the inhibitory effects of these compounds were highly dependent on the inhibitor concentration and that the drug metabolic reactivity was higher than that in human hepatocytes. These findings suggested that the TC-HepG2 cell model was useful in DDI assessment.

One mechanism of drug hepatotoxicity is metabolic activation. The report by a National Institutes of Health group in the United States, which reported on reactive metabolites of the antipyretic analgesic acetaminophen in the 1970s, is the first to highlight the significance of the assessment of toxic metabolic intermediates of drugs [50]. Other examples of pharmaceutical-induced hepatotoxicity caused by metabolic activation include the diabetes drug troglitazone and the prostate cancer drug flutamide [51–55]. All these compounds are metabolically activated by CYPs, which is the main enzyme responsible for drug metabolism [17, 56]. The toxicity of these compounds was impossible to detect at the nonclinical trial stage, and their toxicity profiles were only discovered after their launch. Particularly, troglitazone caused death early after its launch, leading to its discontinuation [57]. Hepatotoxicity can result from the metabolic activation of the causative compound in some cases; therefore, to investigate whether the TC-HepG2 cell model was able to detect hepatotoxicity, we tested the cell toxicity profiles of two compounds whose toxicities were shown to result from their metabolism by CYPs. The aflatoxin B1 forms DNA adduct as a result of metabolic activation induced mainly by CYP3A4 [37]. This results in the DNA replication inhibition, which manifests as cytotoxicity. Sterigmatocystin is a biosynthesized intermediate of aflatoxin B1 and was shown to exert toxicity via the same mechanism as aflatoxin B1 [38]. The results presented in the current study indicated that both compounds led to a concentration-dependent decrease in the survival of TC-HepG2 cells (Fig 7), whereas neither compound was toxic at the same concentrations in parental HepG2 cells lacking CYPs. These results suggest that the TC-HepG2 cells could be utilized to assess cytotoxicity of active metabolites of compounds.

The MAC system was successfully used to establish the TC-HepG2 cell model. In addition to those mentioned above, the other merit of the MAC system is the flexibility with which genetic modifications can be introduced at any stage of the construction. To this end, multiple gene integrations into the MAC can be achieved via a multiple integrase system [58] or via simultaneous/sequential integration systems [59]. These genetic modification techniques theoretically allow an infinite number of genetic modifications to be introduced to MACs, which are then transferred into various host cells and continuous refinements. Thus, although only four CYPs were introduced in the current study, if needed, this approach allows for the addition of other genes, which can significantly decrease both the cost and time required for research. This stable gene transfer method allows not only the transfer of genes used in the current study but also the retransfer of several other pharmacokinetics-related genes. Overall, this streamlined method permits the establishment of cell-based models which can exhibit the nearly identical drug metabolism and pharmacokinetics profiles of human cells that can be refined further. In summary, the TC-HepG2 cells generated and characterized in the current study can provide a highly versatile model for use during the early stages of drug discovery, which require a high degree of processing capacity and reliability.

## Supporting information

S1 Fig PAC construction of the 4CYPs-POR. (A) Map of the 4CYPs-POR cassette constructed in a PAC vector. The expression cassette comprised cDNAs for *CYP2C9*, *CYP2C19*, *CYP2D6*, *CYP3A4*, and *POR*, each under the control of a CAG promoter and flanked with HS4 insulators. The 4CYPs-POR cassette was followed by exons 3–9 of human *HPRT* gene and a loxP site. (B) Confirmation of the PAC construction by PCR.
(TIF)

S2 Fig Image of 4CYPs-POR PAC loading to the MAC6 in CHO cells. (TIF)

S3 Fig Expression of endogenous control genes. Each column represents the mean ± S.E. (*n* = 3). Each gene expression in HepG2 cells as a control revealed 100%.
(TIF)
