# Supplementary figures and images for: Establishment of a novel hepatocyte model that expresses four cytochrome P450 genes stably via mammalian-derived artificial chromosome for pharmacokinetics and toxicity studies
Source: PLoS One. 2017 Oct 24;12(10):e0187072. doi: 10.1371/journal.pone.0187072 (PMC5655360; doi:10.1371/journal.pone.0187072)

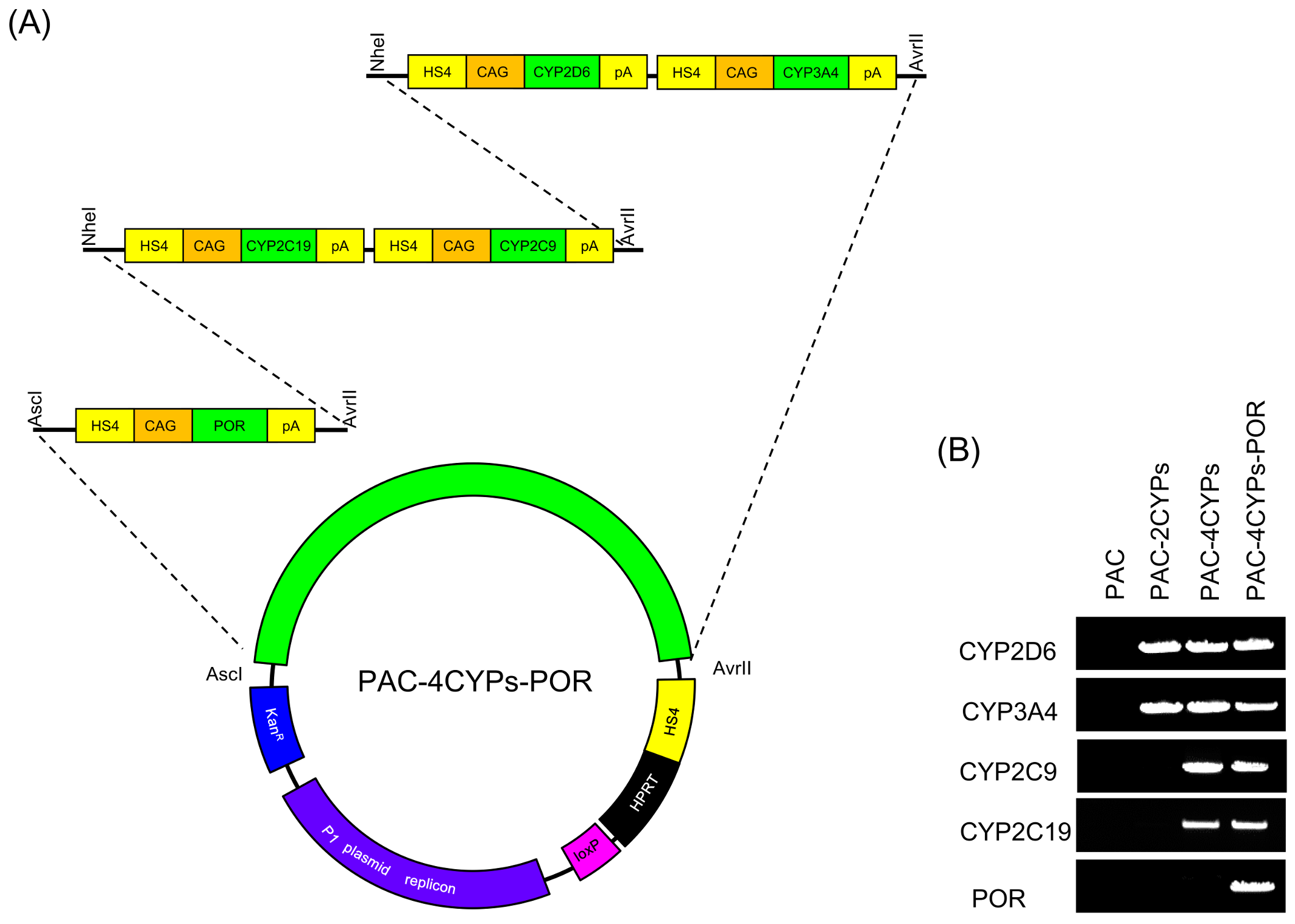

Supplement: S1 Fig — (A) Map of the 4CYPs-POR cassette constructed in a PAC vector. The expression cassette comprised cDNAs for CYP2C9, CYP2C19, CYP2D6, CYP3A4, and POR, each under the control of a CAG promoter and flanked with HS4 insulators. The 4CYPs-POR cassette was followed by exons 3–9 of human HPRT gene and a loxP site. (B) Confirmation of the PAC construction by PCR. (TIF) [file pone.0187072.s001.tif]

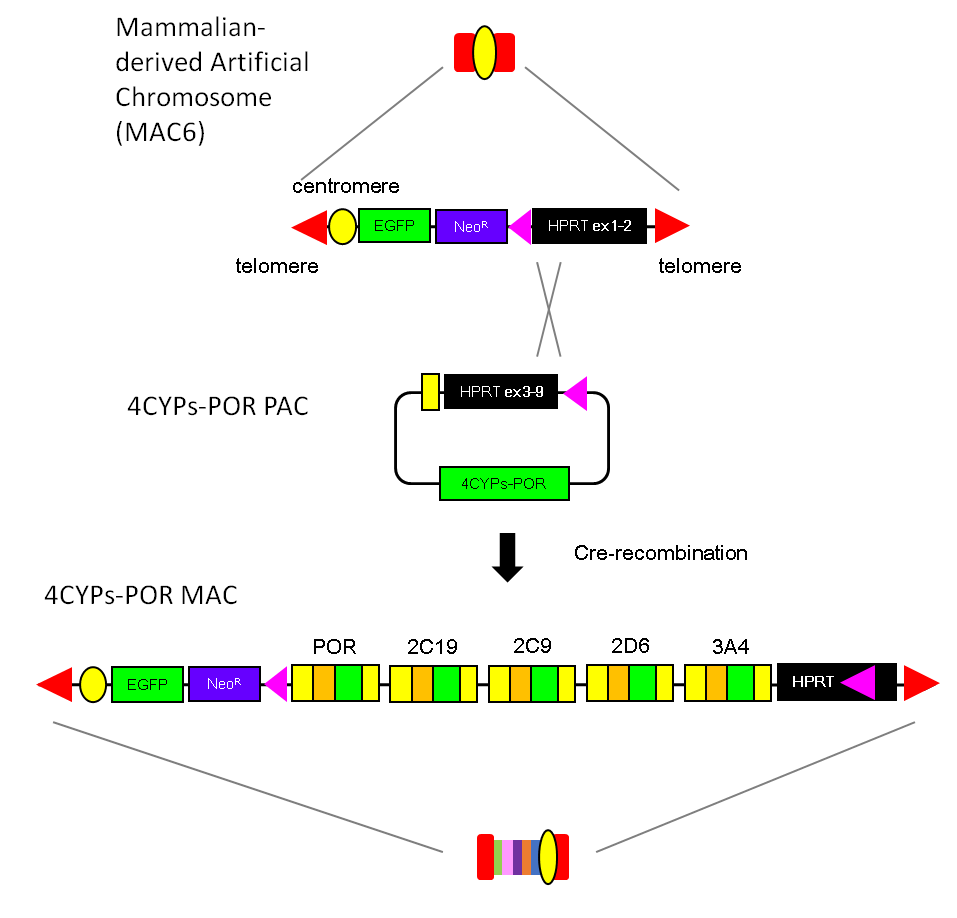

Supplement: S2 Fig — (TIF) [file pone.0187072.s002.tif]

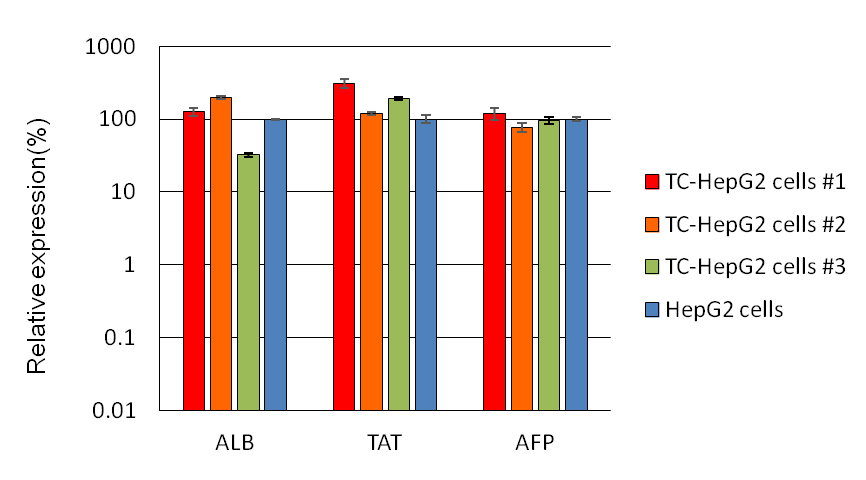

Supplement: S3 Fig — Each column represents the mean ± S.E. (n = 3). Each gene expression in HepG2 cells as a control revealed 100%. (TIF) [file pone.0187072.s003.tif]
